# Induction of kidney tumours in the rats by feeding Encephalartos hildebrandtii for short periods.

**DOI:** 10.1038/bjc.1969.93

**Published:** 1969-12

**Authors:** G. M. Mugera

## Abstract

**Images:**


### INDUCTION OF KIDNEY TUMOURS IN THE RATS BY FEEDING *ENCEPHALARTOS HILDEBRANDTII* FOR SHORT PERIODS

G. M. MUGERA

*From the Department of Veterinary Pathology and Microbiology, University College, Nairobi, P.O. Kabete, Kenya*

Received for publication June 5, 1969

THE production of tumours in liver, kidney and lungs following chronic feeding of crude meal prepared from nuts of *Encephalartos hildebrandtii* has been reported by Mugera and Nderito (1968). The purpose of this paper is to report the findings of short-term feeding experiments.

### MATERIALS AND METHODS

*Encephalartos hildebrandtii* flour was prepared as described in the previous paper (Mugera and Nderito, 1968). The basal diet consisted of commercially available chicken mash, on which excellent growth of the control animals was obtained. The *Encephalartos hildebrandtii* flour was thoroughly mixed with the basal diet to give a 5 per cent concentration and fed to rats *ad libitum*. These were male and female weanling rats bred at Kabete. All animals had free access to water. The rats were divided into 6 groups of 20 rats. Group I was given the experimental diet for 28 days, Group II for 21 days, Group III for 14 days, Group IV for 7 days, Group V for 4 days and Group VI was fed the control diet. The rats were returned to the basal diet for observation. Necropsies were carried out on all animals which died naturally and on those killed 18 months after start of the experiment.

### RESULTS

Seven rats died between 6 and 9 months after the start of the experiment, 4 from Group I and 3 from Group II. No tumours were found in any of these rats. A summary of the experiment in which development of tumours was observed is given in Table I.

TABLE I

| Group | No. of rats with tumour | Tumours | |
|---|---|---|---|
| | | Liver | Kidney |
| I | 16 | 5 | 16 |
| II | 15 | 3 | 15 |
| III | 18 | 0 | 18 |
| IV | 12 | 0 | 12 |
| V | 0 | 0 | 0 |
| VI | 0 | 0 | 0 |

Sixty-one of the 100 rats fed the experimental diet had tumours in one or both kidneys and a total of 107 kidneys had tumorous growths. The majority of the 107 tumours could be classified; 40 were adenomas, 31 fibrosarcoma, 22 nephroblastomas and 7 carcinomas. The adenomas and fibrosarcomas were similar to those described by Mugera and Nderito (1968) in rats after chronic feeding of the

*Encephalartos hildebrandtii* flour. The nephroblastomas had rows of epithelial cells surrounded by undifferentiated cells. In some areas of these tumours the epithelial cells were surrounded by smooth muscle cells and other areas resembled fibrosarcoma without epithelial cells. The nephrobastomas formed large masses (Fig. 1) and could not be differentiated grossly from carcinomas (Fig. 4).

Carcinomas were also large, usually affecting only one kidney. The tumour masses occupied much of the abdominal cavity and were usually adherent to the adjacent organs or implanted tumour masses were seen on the wall of the abdominal cavity or peritoneum (Fig. 4). Histologically these tumours were composed of large pale-staining cells with large nuclei. The nuclei were vesicular and had marked concentration of the chromatin at the nuclear membrane. They had one or more large nucleoli. The cells were arranged in solid masses or cords and there were no tubular formations.

Neoplasms of the liver were found in 8 rats only, 6 were cystadenomas, 3 bile duct adenomas and 2 hepatomas. Hyperplastic changes in the mucosa of the urinary bladder and pelvis of the kidney were noted in 12 rats ranging from focal epithelial thickening to papillary structures.

### DISCUSSION

Seven days was the shortest period of feeding *Encephalartos hildebrandtii* flour to rats to induce kidney tumours. The production of large numbers of renal tumours and the correspondingly low incidence of hepatic neoplasms in rats after short exposure to *Encephalartos hildebrandtii* flour closely resemble the observations made by Laqueur (1964) with *Cycas circinalis* meal and cycasin and by Magee and Barnes (1962) with nitrosamines. These findings suggest that the carcinogenic factor in *Encephalartos hildebrandtii* may be cycasin.

### SUMMARY

Tumours of the kidney developed in rats after short exposure to *Encephalartos hildebrandtii*. The tumours resembled those seen in rats after chronic feeding of the same flour, with the exception of nephroblastoma which was noted with short exposure but not with chronic feeding. The shortest period of exposure capable of inducing kidney tumours was 7 days.

### REFERENCES

Laqueur, G. L.—(1964) *Fedn Proc. Fedn Am. Socs exp. Biol.*, **23**, 1386.
Magee, P. N. and Barnes, J. M.—(1962) *J. Path. Bact.*, **84**, 19.
Mugera, G. M. and Nderito, P.—(1968) *Br. J. Cancer*, **22**, 563.

---

### EXPLANATION OF PLATES

Fig. 1.—A rat from Group I, killed 18 months after the start of the experiment, showing a nephroblastoma of the left kidney and the normal right kidney.

Fig. 2.—A histological section of the tumour shown in Fig. 1. There is an adenomatous area at top right, blood filled cavities in the left-hand lower corner and the fibrous component of the tumour between.

Fig. 3.—Another area from the tumour illustrated in Fig. 2 showing smooth muscle cells in the lower left-hand half of the field and fibrous tissue in the other half.

Fig. 4.—An adenocarcinoma of a kidney of a rat from Group II killed 18 months after the start of the experiment. There is a white mass of tumour implanted on the wall of the abdominal cavity visible at the left-hand end of the photograph.

Fig. 5.—Histological section of the tumour shown in Fig. 4.


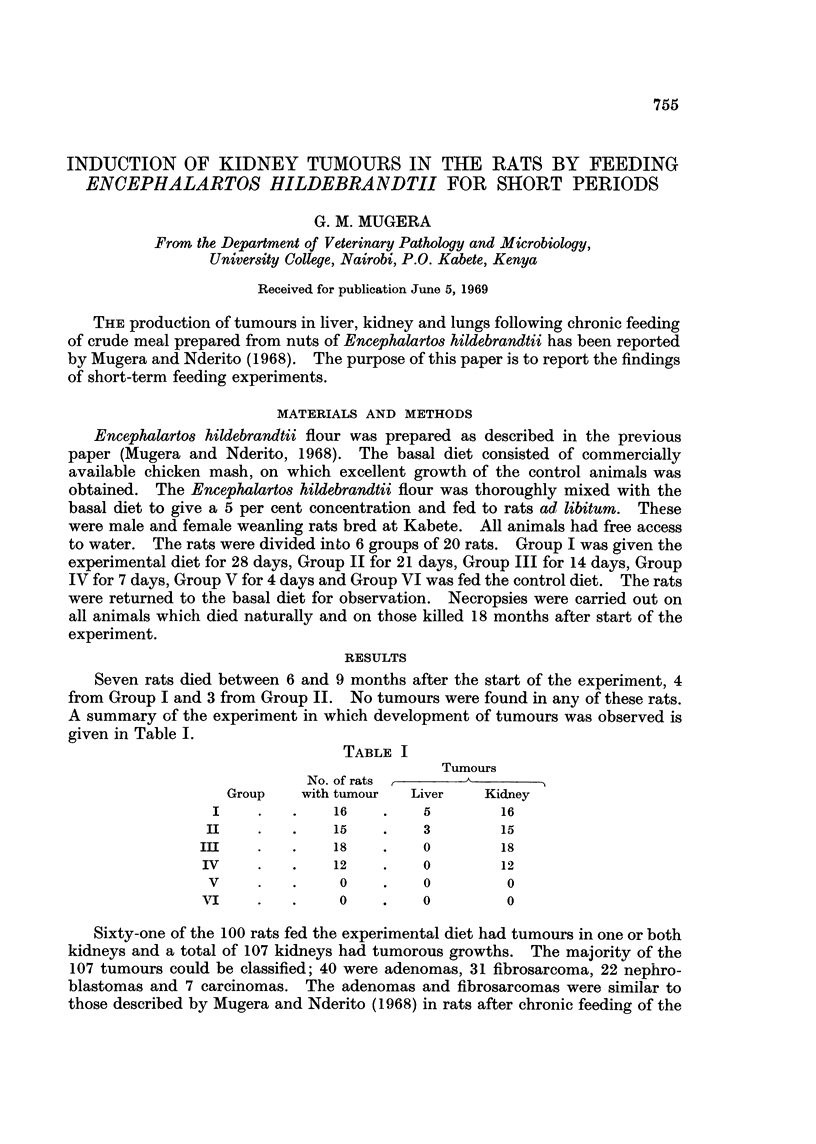

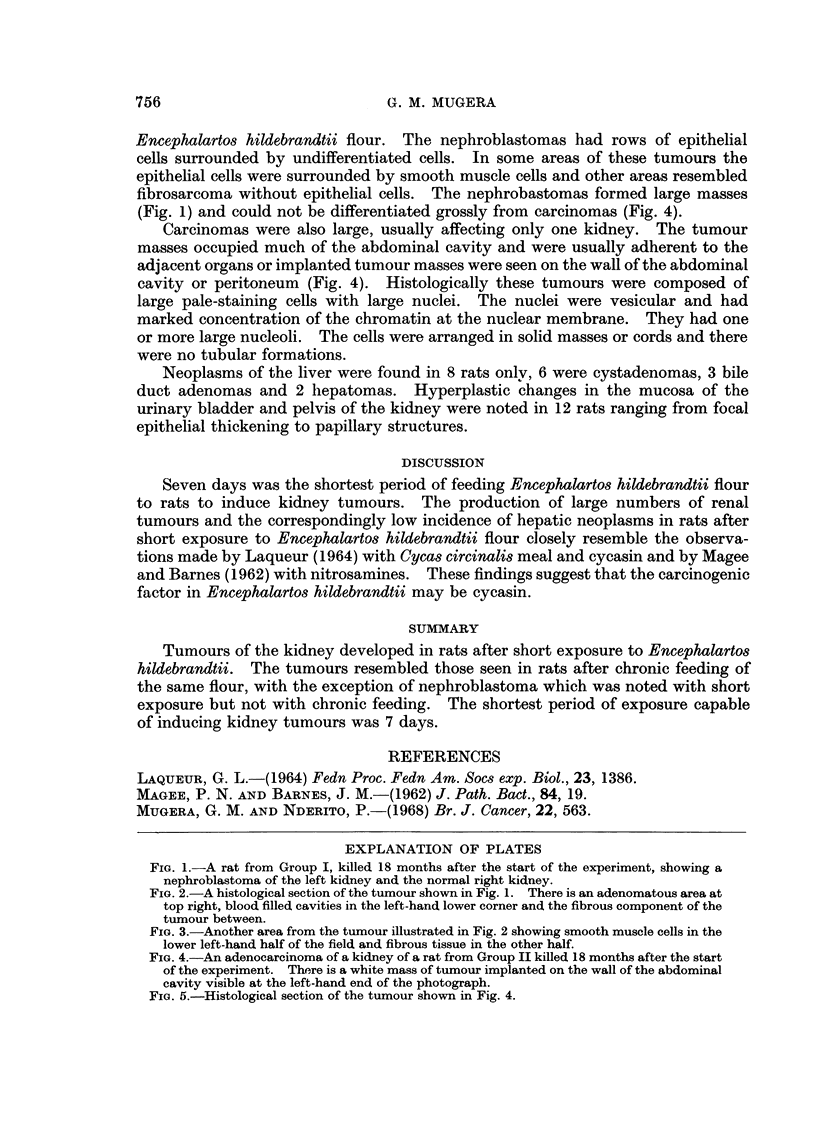

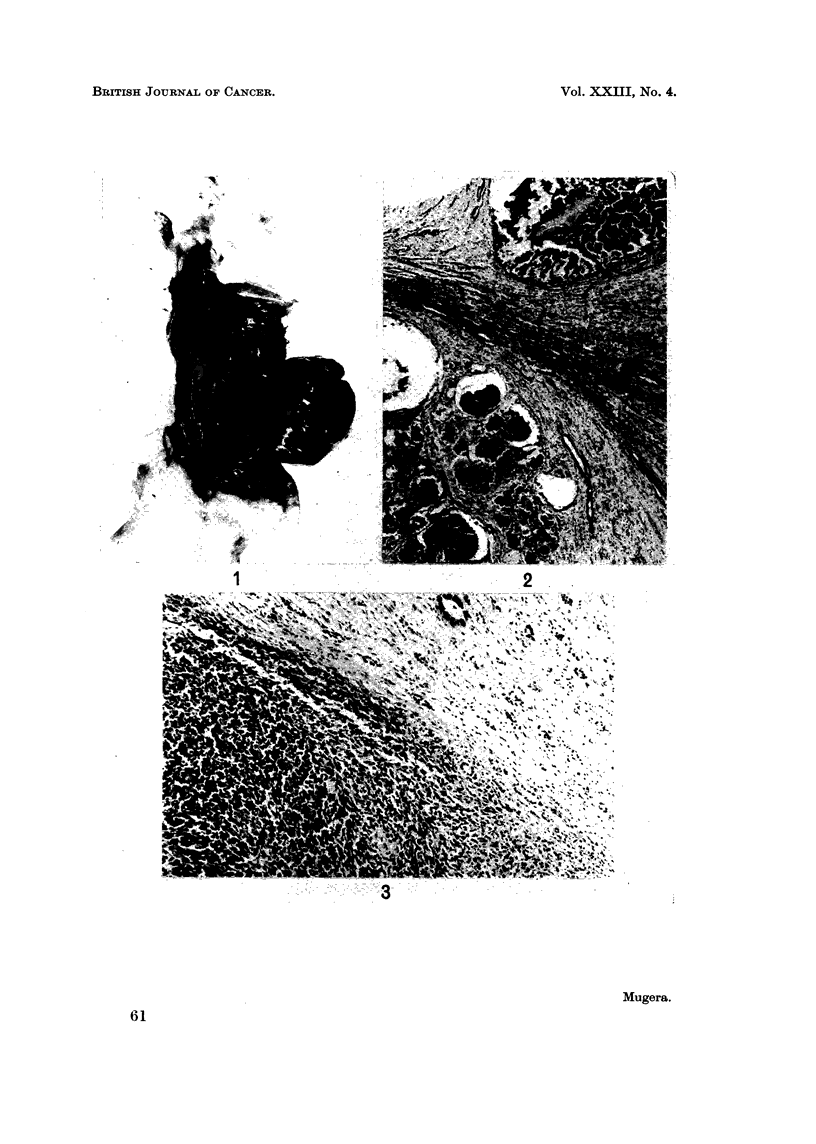

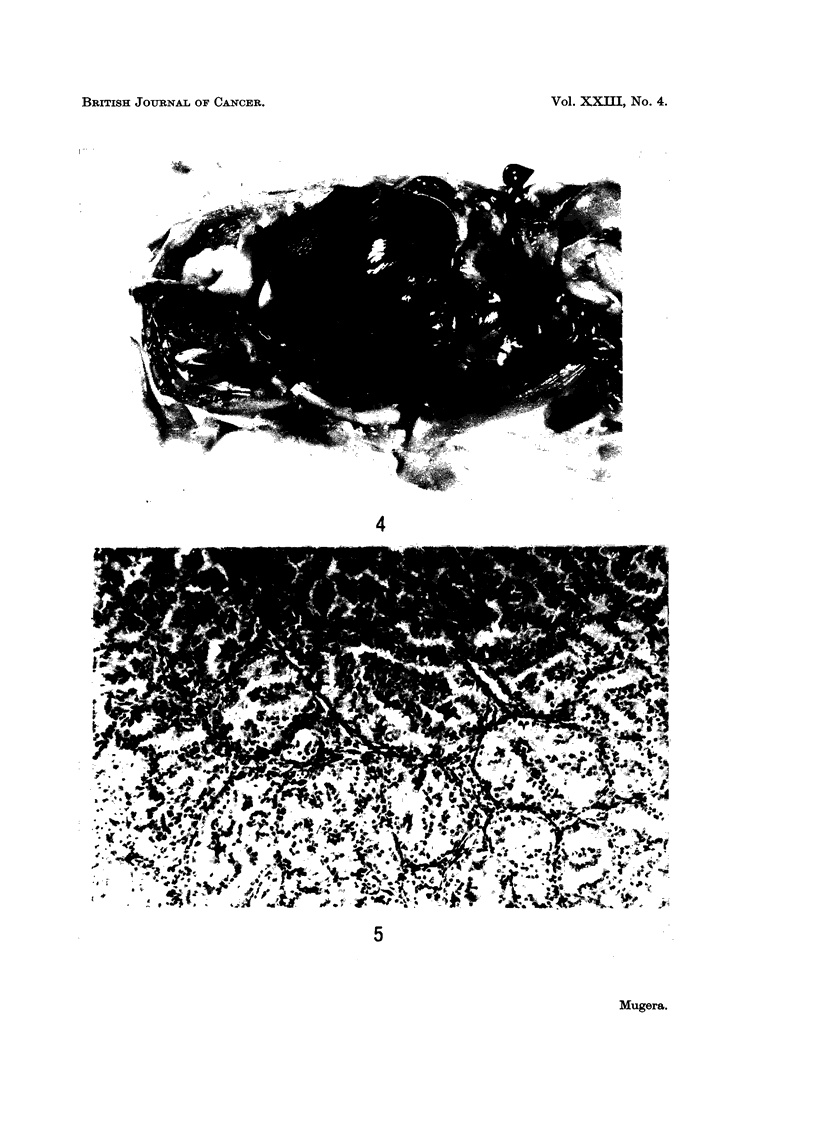


## References

[OCR_00731] AL-HINDAWI A. Y., WILSON G. M. (1965). THE EFFECT OF IRRADIATION ON THE FUNCTION AND SURVIVAL OF RAT THYROID CELLS.. Clin Sci.

[OCR_00733] CROOKS J., GREIG W. R., MACGREGOR A. G., MCINTOSH J. A. (1964). A QUANTITATIVE METHOD OF MEASURING THE EFFECTS OF X IRRADIATION ON THE GROWTH AND FUNCTION OF THE RAT THYROID GLAND.. Br J Radiol.

[OCR_00739] DONIACH I., FRANCOIS P. E. (1960). Comparison of iodine-124 and iodine-131 in inhibition of goitrogenesis in the rat.. Nature.

[OCR_00741] DONIACH I., LOGOTHETOPOULOS J. H. (1955). Effects of radioactive iodine on the rat thyroid's function, regeneration and response to goitrogens.. Br J Cancer.

[OCR_00747] FELLER D. D., CHAIKOFF I. L. (1949). The changes induced in iodine metabolism of the rat by internal radiation of its thyroid with I131.. Endocrinology.

[OCR_00751] Gibson J. M., Doniach I. (1967). Correlation of dose of x-radiation to the rat thyroid gland with degree of subsequent impairment of response to goitrogenic stimulus.. Br J Cancer.

[OCR_00757] Greig W. R., McInnes J. (1966). Radioprotection of the rat thyroid by different antithyroid compounds.. Br J Radiol.

[OCR_00759] Hempelmann L. H., Pifer J. W., Burke G. J., Terry R., Ames W. R. (1967). Neoplasms in persons treated with x rays in infancy for thymic enlargement. A report of the third follow-up survey.. J Natl Cancer Inst.

[OCR_00105] LAQUEUR G. L. (1964). CARCINOGENIC EFFECTS OF CYCAD MEAL AND CYCASIN, METHYLAZOXYMETHANOL GLYCOSIDE, IN RATS AND EFFECTS OF CYCASIN IN GERMFREE RATS.. Fed Proc.

[OCR_00763] MALOOF F., DOBYNS B. M., VICKERY A. L. (1952). The effects of various doses of radioactive iodine on the function and structure of the thyroid of the rat.. Endocrinology.

[OCR_00108] Mugera G. M., Nderito P. (1968). Tumours of the liver, kidney and lungs in rats fed Encephalartos hildebrandtii.. Br J Cancer.

